# Socioeconomic deprivation in early life and symptoms of depression and anxiety in young adulthood: mediating role of hippocampal connectivity

**DOI:** 10.1017/S0033291720004754

**Published:** 2022-10

**Authors:** Pavla Čermaková, Lenka Andrýsková, Milan Brázdil, Klára Marečková

**Affiliations:** 1 Third Faculty of Medicine, Charles University Prague, Prague, Czech Republic; 2 National Institute of Mental Health, Klecany, Czech Republic; 3 Second Faculty of Medicine, Charles University Prague, Prague, Czech Republic; 4 Faculty of Science, RECETOX, Brno, MU, Czech Republic; 5 Brain and Mind Research, Central European Institute of Technology, Masaryk University, Brno, Czech Republic

**Keywords:** depression, anxiety, fMRI, sex differences

## Abstract

**Background:**

Experience of early-life socioeconomic deprivation (ELSD) may increase the risk of mental disorders in young adulthood. This association may be mediated by structural and functional alterations of the hippocampus.

**Methods:**

We conducted a prospective cohort study on 122 participants of the European Longitudinal Study of Pregnancy and Childhood. Information about ELSD was collected via questionnaire from mothers during the first 18 months of participants’ lives. At age 23–24, participants underwent examination by structural magnetic resonance imaging, resting-state functional connectivity and assessment of depressive symptoms (Mood and Feelings Questionnaire) and anxiety (Spielberger State-Trait Anxiety Inventory). The association of ELSD with brain outcomes in young adulthood was assessed with correlations, linear regression (adjusting for sex, socioeconomic position and mother's mental health) and moderated mediation analysis.

**Results:**

Higher ELSD was associated with greater depressive symptoms (*B* = 0.22; *p* = 0.001), trait anxiety (*B* = 0.07; *p* = 0.02) and lower global connectivity of the right hippocampus (*B* = −0.01; *p* = 0.02). These associations persisted when adjusted for covariates. In women, lower global connectivity of the right hippocampus was associated with stronger trait anxiety (*B* = −4.14; *p* = 0.01). Global connectivity of the right hippocampus as well as connectivity between the right hippocampus and the left middle temporal gyrus mediated the association between ELSD and trait anxiety in women. Higher ELSD correlated with a lower volume of the right hippocampus in men, but the volume of the right hippocampus was not related to mental health.

**Conclusions:**

Early preventive strategies targeted at children from socioeconomically deprived families may yield long-lasting benefits for the mental health of the population.

## Introduction

Foundations for good mental health of an individual are established during the first 1000 days of life (Chen & Miller, 2012; Cusick & Georgieff, 2016). The rapid growth of neurons and synapses (Rice & Barone, 2000) makes the brain particularly vulnerable to early-life adversities. For example, individuals who grow up in households that have difficulties paying for accommodation, food, clothes and basic utilities may have lower access to resources that could enable them to fully develop their potential. Experience of such early-life socioeconomic deprivation (ELSD) may leave a mark on the developing brain of the offspring in the form of reductions in grey and white matter volumes (Brito & Noble, 2014), leading to a dysregulated physiological stress response and increased vulnerability to anxiety and depression (Mareckova, Klasnja, Andryskova, Brazdil, & Paus, 2019a; Mareckova et al., 2019b). Individuals who faced ELSD exhibit smaller volumes of hippocampus (Staff et al., 2012), which, besides its function in memory and learning, plays a role in the regulation of stress, motivation and emotions. Lower hippocampal size is suggested to be involved in the emergence of depression (Rao et al., 2010) and anxiety (Gorka, Hanson, Radtke, & Hariri, 2014).

The structural abnormalities of the hippocampus are also reflected in its functional alterations, such as its functional connectivity (Suzuki et al., 2013). Hippocampus is connected with diffuse cortical and subcortical regions, for example, positively with amygdala, other regions in the medial and anterior temporal lobe and ventral medial prefrontal cortex, and negatively with dorsal prefrontal and parietal cortex (Barch et al., 2016; Cao et al., 2012; De La Plata et al., 2011). Evidence on the direction of the association between hippocampal volume and connectivity is mixed, with studies reporting both positive and negative relationships, depending on the studied region (Suzuki et al., 2013). Both increased and decreased global and regional functional connectivity of the hippocampus were observed in studies on depression and anxiety (Cao et al., 2012; Cha et al., 2016; Chen & Etkin, 2013; Suzuki et al., 2013). Recently, the experience of ELSD was found associated with lower connectivity between the left hippocampus and the right posterior cingulate cortex and higher connectivity between the left hippocampus and the right fusiform and superior frontal gyrus as well as between the right hippocampus and the right superior frontal gyrus (Barch et al., 2016).

Depression and anxiety are unequally distributed in the world, with higher burden in women and socioeconomically less developed countries (Horackova et al., 2019). In Europe, particularly high prevalence, associated disability and great treatment gap are known for post-communist countries in Central and Eastern Europe (CEE) (Horackova et al., 2019), where also the experience of ELSD is higher than in other regions (Cermakova, Formanek, Kagstrom, & Winkler, 2018). As three-quarters of lifetime mental disorders appear by age 24 (Kessler et al., 2005), young adulthood is an important life stage to study their antecedents. We aimed to determine whether ELSD predicts symptoms of depression and anxiety in young adults residing in a CEE country and to find potential biomarkers of these associations. We hypothesized that higher ELSD is associated with greater symptoms of depression and anxiety, lower hippocampal volume and lower connectivity in young adulthood and that hippocampal volume and/or connectivity mediate the association of ELSD with mental health.

## Methods and materials

### Participants

We studied 131 young adults from the Czech Republic who participated in the European Longitudinal Study of Pregnancy and Childhood (ELSPAC-CZ) (Piler et al., 2017) and its neuroimaging follow-up Biomarkers and Underlying Mechanisms of Vulnerability to Depression (VULDE). ELSPAC-CZ is a prenatal cohort (*n* = 5151) whose members were born between 1991 and 1992; their mothers were enrolled between the ultrasound examination at 20th week of pregnancy and the birth of the child. The parents were asked to fill in several sets of questionnaires regarding themselves and their child, with the first set being introduced upon the enrolment to the study and the last set when their child reached the age of 19.

The VULDE neuroimaging follow-up was conducted on a sub-group of the ‘ELSPAC children’ (*n* = 131, 53% women) aged 23–24 years (see also online Supplementary Methods 1.1 and Fig. S1). They underwent extensive magnetic resonance imaging (MRI) of the brain using 3T Siemens Prisma MRI scanner, including structural MRI [T1-weighted MPRAGE images of the whole brain acquired with 64 channel head/neck coil; voxel size 1 × 1 × 1 mm, repetition time (TR) 2300 ms, echo time (TE) 2.34 ms, inversion time (TI) 900 ms, flip angle 8 degrees], a 7 min closed-eyes resting-state functional MRI (fMRI) (voxel size 3 × 3 × 3 mm, TR 2080 ms, TE 30 ms, flip angle 90 degrees, 39 slices, matrix 64 × 64, 200 measurements), together with measures on their mental health. From 131 participants in the VULDE study, two did not undergo fMRI and seven had missing data on ELSD, leaving the final sample of 122 individuals. All participants provided written informed consent and ethical approval was obtained from the ELSPAC Ethics Committee.

### ELSD

Information about ELSD was collected as a part of the original ELSPAC-CZ. ELSD was assessed by questionnaires administered to the mother at 6 and 18 months of the offspring. Mothers used a four-point Likert scale to answer how difficult it is to secure the family with the following five things: food, clothes, heating, rent/other fees and things necessary for the child. The final ELSD score was calculated as the mean of the scores for 6 and 18 months, with higher values indicating more severe deprivation (see also online Supplementary Methods 1.2).

### Mental health in young adulthood

Information about mental health was collected as a part of VULDE. Depressive symptoms were assessed using the Mood and Feelings Questionnaire (Angold & Costello, 1987), which includes 33 questions answered on a three-point Likert scale. Anxiety was measured using the Spielberger State-Trait Anxiety Inventory (Spielberger & Gorsuch, 1983). Here we utilize trait anxiety as we hypothesized that ELSD will be associated with enduring changes in personality rather than transient anxiety state. Trait anxiety was assessed with 20 questions, rated on a four-point Likert scale.

### Covariates

Data on covariates were collected as a part of ELSPAC-CZ. To determine the independent association of ELSD with outcomes in young adulthood, early-life covariates were accounted for in two steps. Model 1 was adjusted for sex and covariates related to early-life socioeconomic position (SEP): mother's education, father's education, father's occupation, household income, basic utilities, household items and crowding ratio. Model 2 was adjusted for sex and covariates related to mother's mental health: mother's depression and anxiety (see also online Supplementary Methods 1.3).

### Statistical analysis

#### Association of ELSD with mental health in young adulthood

The relationship between ELSD and depressive symptoms/trait anxiety in young adulthood was assessed with linear regression and is expressed as *B* with 95% confidence intervals (CI). The *p* values were adjusted for false discovery rate (FDR) using the Benjamini–Hochberg method. As previously reported (Horackova et al., 2019; Orbach, Herzog, & Fritz, 2019), the measures of depressive symptoms and trait anxiety were skewed. When using the original variables in linear regression, the assumption of normal distribution of residuals was not met. After log-transformation, the residuals were normally distributed; therefore, log-transformed depressive symptoms and trait anxiety were used. Then, the association was adjusted for sex and covariates related to early-life SEP (Model 1) and sex and covariates related to mother's mental health (Model 2). Collinearity was assessed with variance inflation factor (VIF). As the VIF was lower than 3 for all covariates in both models, all covariates were used. Moderation by sex was tested by including an interaction term (ELSD × sex) into the unadjusted model and stratified analyses were performed, where appropriate.

#### Analysis of hippocampal volume

The right and left hippocampal volumes and their subfields were calculated using Freesurfer version 6.0 according to a previously described method (Mareckova et al., 2018; see also online Supplementary Methods 1.4). The hippocampal subfields were left and right parasubiculum, presubiculum, subiculum, cornu ammonis (CA) 1, CA2/3, CA4, granule cell layer of the dentate gyrus (GC/DG), hippocampal-amygdaloid transition area, fimbria, molecular layer, hippocampal fissure and hippocampal tail. Volumes of left and right hippocampus and hippocampal subfields were corrected for total brain volume (as brain size, excluding cerebrospinal fluid) and regressed out from the hippocampal volume.

The associations of the right and left hippocampal volume with ELSD as well as mental health in young adulthood were assessed with linear regression, moderation by sex was assessed. A full factorial general linear model (GLM) explored the impact of ELSD, sex and type of hippocampal subfield on hippocampal volume. Stratified analyses were conducted, where appropriate. The associations were adjusted for FDR (see online Supplementary Methods 1.6).

#### Analysis of hippocampal connectivity

CONN Functional Connectivity Toolbox version 18.b. and its default pre-processing pipeline (Whitfield-Gabrieli & Nieto-Castanon, 2012, see also online Supplementary Methods 1.5) was used for functional connectivity analysis, which was conducted in two stages: First, global hippocampal connectivity was assessed by a seed-to-voxel analysis, and second, regional hippocampal connectivity with a post-hoc region of interest (ROI)-to-ROI analysis.

Seed-to-voxel analysis assessed functional connectivity between the seeds of interest (right and left hippocampus) and the rest of the brain, the seeds having been selected based on Harvard-Oxford Structures Atlas (http://fsl.fmrib.ox.ac.uk/fsl/fslwiki/Atlases). Pearson's correlation coefficients were calculated between the seed time course and the time course of all other voxels in the brain. Seed-to-voxel results are reported when significant at a voxel-wise threshold level of *p* < 0.001 uncorrected and a cluster-level threshold of *p* < 0.05 FDR-corrected.

The correlation coefficients were converted to normally distributed scores using Fisher's transformation. The coefficients for clusters of voxels showing a significant correlation with the left and/or the right hippocampus were used as dependent variables in further analyses. Linear regression was performed to assess associations between ELSD and the significant clusters, adjusting for covariates (Models 1 and 2). Moderation by sex was tested in the unadjusted model.

A post-hoc ROI-to-ROI analysis evaluated correlations between the hippocampus and specific ROIs. The brain was parcelled on the Harvard-Oxford atlas (cortical and subcortical areas) and Automated Anatomical Labelling atlas (cerebellar areas) and connectivity between the hippocampus and all the ROIs was calculated. Higher *z*-scores indicated positive correlations between ROIs (increased signal time series synchronization indicating higher functional connectivity), lower *z*-scores negative correlations (decreased synchronicity between ROIs showing lower connectivity). Fisher's transformation was applied to all *z*-scores, and correlation coefficients were converted into standard scores. A *p* value corrected for FDR <0.05 identified statistically significant correlations between the hippocampus and the ROIs.

#### Moderated mediation analysis

To be considered as a mediator, the variable of interest needs to be associated with both ELSD and a mental health outcome in young adulthood. As we observed moderation by sex in several of our analyses, we hypothesized that the mediation between the ELSD and a mental health outcome in young adulthood may depend on sex. A moderated mediation analysis, which is a suggested method to explore this question (Hayes, 2018), therefore evaluated a potential role of hippocampal volume/connectivity as a mediator between ELSD and mental health in young adulthood, conditional on sex. The analysis was carried out using the PROCESS macro (Hayes, 2018) for SPSS. Conditional indirect effects were assessed for significance using bootstrapped bias-corrected 95% CI constructed around indirect effect estimates, based on 10 000 bootstrapping iterations.

## Results

### Association of ELSD with mental health in young adulthood

We studied 122 individuals (52% women; characteristics presented in Table 1). Higher ELSD was associated with more depressive symptoms (*B* = 0.22; 95% CI 0.10–0.34; *p* = 0.001/*p*-FDR = 0.002). The associations persisted, when adjusted for sex and early-life SEP (Model 1: *B* = 0.26; 95% CI 0.10–0.41; *p* = 0.002) or sex and mother's mental health (Model 2: *B* = 0.29; 95% CI 0.14–0.44; *p* < 0.001). Sex moderated the association (*p* for interaction 0.03); stratified analyses showed its presence only in women (*B* = 0.38; 95% CI 0.18–0.59; *p* < 0.001) and not men (*B* = 0.11; 95% CI −0.05 to 0.26; *p* = 0.18). Similarly, higher ELSD was associated with a stronger trait anxiety (*B* = 0.07; 95% CI 0.01–0.12; *p* = 0.02/*p*-FDR = 0.02) and the association persisted after adjustment for covariates (Model 1: *B* = 0.08; 95% CI 0.02–0.15; *p* = 0.02; Model 2: *B* = 0.08; 95% CI 0.02–0.15; *p* = 0.02), but sex was not a moderator (*p* for interaction 0.36). Online Supplementary Table S1 presents correlations between main measures and online Supplementary Table S2 shows associations of covariates with mental health in young adulthood.

**Table 1.** Characteristics of study participants (*n* = 122)

Characteristic	Value	*N*
Women, *n* (%)	63 (52)	122
Socioeconomic deprivation in early life, median (IQR)	2.5 (3.5)	122
Depressive symptoms in young adulthood, median (IQR)	7.5 (8)	122
Anxiety trait in young adulthood, median (IQR)	27 (14)	122
Covariates related to early-life socioeconomic position		
Mother's education: university or postgraduate, *n* (%)	44 (45)	98
Father's education: university or postgraduate, *n* (%)	52 (52)	100
Father's occupation: ‘white-collar’ worker^a^, *n* (%)	75 (62)	120
Household income in CZK, median (IQR)	7083 (2875)	120
Basic utilities, median (IQR)	7 (2)	122
Household items, mean ± s.d.	6 ± 1	98
Crowding ratio, mean ± s.d.	1.7 ± 0.6	122
Covariates related to mental health		
Mother's depression, median (IQR)	6 (5)	100
Mother's anxiety, mean ± s.d.	6.5 ± 4.1	103

IQR, interquartile range; s.d., standard deviation.

a White-collar worker defined as class I, II and III according to Erikson, Goldthorpe and Portocareros (EGP) scheme (Erikson, Goldthorpe, & Portocarero, 1979).

### Hippocampal volume

ELSD was not associated with the right hippocampal volume (*B* = −16.75; 95% CI −62.56 to 29.06; *p* = 0.47/*p*-FDR = 0.94), but sex was a moderator (*p* for interaction 0.01) and stratified analysis showed an association in men (*B* = −71.04; 95% CI −136.03 to −6.05; *p* = 0.03), but not women (*B* = 48.66; 95% CI −13.40 to 110.72; *p* = 0.12). However, after adjustment for early-life SEP (Model 1: *B* = −61.13; 95% CI −143.84 to 21.59; *p* = 0.14) and mother's mental health (Model 2: *B* = −32.96; 95% CI −124.30 to 58.39; *p* = 0.46), the association in men did not persist. A full-factorial GLM revealed an interaction between ELSD, sex and the subfield of the right hippocampus [*F*_(11,108)_ = 2.34, *p* = 0.01]. In post hoc analyses, higher ELSD correlated with lower volumes of six subfields in men: CA1, CA3, CA4, GC/DG, molecular layer and hippocampal fissure, but only the correlation with CA1 persisted after FDR correction (online Supplementary Table S3). However, when adjusted for total right hippocampal volume, ELSD was no longer statistically associated with CA1 (*p* = 0.60; not presented in tables). Correlations between individual hippocampal subfield volumes are presented in online Supplementary Table S4. As the right hippocampal volume was not associated with any mental health outcome (depressive symptoms: *B* = 0.00; 95% CI 0.00–0.01; *p* = 0.11/*p*-FDR = 0.15; trait anxiety: *B* = 0.00; 95% CI 0.00–0.00; *p* = 0.57/*p*-FDR = 0.57), we did not proceed with mediation analysis.

ELSD was not associated with the left hippocampal volume (*B* = −14.11; 95% CI −58.51 to 30.30; *p* = 0.53/*p*-FDR = 0.53) and there was no moderation by sex (*p* for interaction 0.10). Surprisingly, higher left hippocampal volume was associated with greater depressive symptoms and trait anxiety, but the associations did not persist after FDR corrections (depressive symptoms: *B* = 0.001; 95% CI 0.00–0.001; *p* = 0.02/*p*-FDR = 0.08; trait anxiety: *B* = 0.00; 95% CI 0.00–0.00; *p* = 0.04/*p*-FDR = 0.08). A full-factorial GLM did not reveal an interaction between ELSD, sex and the subfield type of the left hippocampus [*F*_(11,108)_ = 0.84, *p* = 0.60]. Correlations between volumes of subfield of the left and the right hippocampus are presented in online Supplementary Table S5.

### Global hippocampal connectivity

**Fig. 1.**
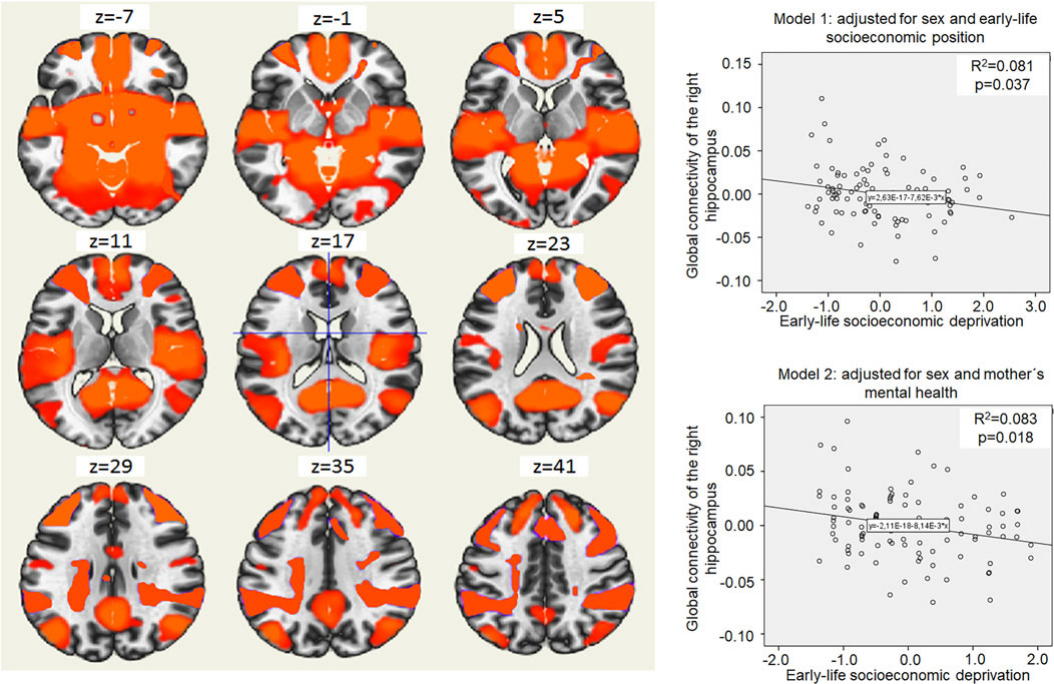
Global connectivity of the right hippocampus and its relation to early-life socioeconomic deprivation. The left side on the figure shows axial brain slices displaying the average global functional connectivity map of the right hippocampus. The right side of the figure shows an association of higher early-life socioeconomic deprivation (*x*-axis) and lower global connectivity of the right hippocampus in young adulthood (*y*-axis). Coordinates (voxels): 75; 109; 80. The cluster of voxels characterizing global connectivity of the right hippocampus included right and left frontal pole, brain stem, precuneus, lateral occipital cortex, right and left temporal pole, right and left superior frontal gyrus, left middle frontal gyrus, lateral occipital cortex, right middle frontal gyrus, right and left lingual gyrus and other regions (see online Supplementary Table S4). Model 1 shows the association of higher early-life socioeconomic deprivation (*x*-axis) with lower global connectivity of the right hippocampus in young adulthood (*y*-axis), adjusted for sex and covariates related to early-life socioeconomic position (mother's education, father's education, father's occupation, household income, basic utilities, household items and crowding ratio). Model 2 shows the association of higher early-life socioeconomic deprivation (*x*-axis) with lower global connectivity of the right hippocampus in young adulthood (*y*-axis), adjusted for sex and covariates related to mother's mental health (mother's depression and mother's anxiety).

Higher ELSD was associated with lower connectivity between the right hippocampus and a large cluster of voxels (*B* = −0.01; 95% CI −0.01 to −0.001; *p* = 0.02; MNI peak coordinates +24; −16; −18; online Supplementary Table S6). Connectivity between the right hippocampus and this cluster will be further on referred as ‘global connectivity of the right hippocampus’. Higher ELSD was associated with lower global connectivity of the right hippocampus even in adjusted models (Fig. 1), but there was no moderation by sex (*p* for interaction 0.30). Global connectivity of the right hippocampus was not associated with depressive symptoms (*B* = −1.43; 95% CI −5.83 to 2.97; *p* = 0.52) and there was no moderation by sex (*p* for interaction 0.07). Global connectivity of the right hippocampus was not associated with trait anxiety (*B* = −0.44; 95% CI −2.35 to 1.48; *p* = 0.65), but sex was a moderator (*p* for interaction 0.01). Stratified analyses showed an association of higher global connectivity of the right hippocampus with lower trait anxiety in women (*B* = −4.14; 95% CI −7.34 to −0.91; *p* = 0.01), but not in men (*B* = 1.21; 95% CI −1.08 to 3.50; *p* = 0.29).

Higher ELSD was also associated with lower connectivity between the left hippocampus and one cluster of voxels (*B* = −0.01; 95% CI −0.03 to −0.0001; *p* = 0.05) that included mainly inferior temporal gyrus (MNI peak coordinates −52; −32; −26; online Supplementary Table S7). This association lost significance after adjustment for covariates. There was no moderation by sex (*p* for interaction 0.29).

### Regional hippocampal connectivity

A post-hoc ROI-to-ROI analysis assessed which ROI from the large cluster connected with the right hippocampus may drive the aforementioned results. Online Supplementary Fig. S3 presents connectivity between the right hippocampus and all ROIs. Higher ELSD significantly correlated with lower connectivity between the right hippocampus and five ROIs; four passed the FDR correction (Table 2): the left middle temporal gyrus (MTG), the right inferior temporal gyrus, the left paracingulate gyrus (all three were positively connected to the right hippocampus) and the right superior frontal gyrus (negatively connected to the right hippocampus). While these five ROIs were derived independently using the ROI approach, they were all part of the cluster identified by the initial global hippocampal connectivity analysis. Higher connectivity between the right hippocampus and the left MTG correlated with lower trait anxiety in women (*r* = −0.35; *p* = 0.006/*p*-FDR = 0.03); there were no correlations with mental health outcomes in men (not presented in tables).

**Table 2.** Connectivity of the right hippocampus to significant regions of interest and its relationship with socioeconomic deprivation in early life

	Right middle temporal gyrus	Left middle temporal gyrus	Right superior frontal gyrus	Right inferior temporal gyrus	Left paracingulate gyrus
Connectivity with the right hippocampus					
*β*	0.14	0.10	−0.09	0.07	0.10
*T* statistics	8.13	6.89	−5.60	4.76	6.98
*p* value	<0.001	<0.001	<0.001	<0.001	<0.001
*p*-FDR	<0.001	<0.001	<0.001	<0.001	<0.001
Correlation between early-life socioeconomic deprivation and connectivity with the right hippocampus					
*r*	−0.229	−0.193	−0.211	−0.231	−0.197
*p* value	0.011	0.033	0.020	0.011	0.030
*p*-FDR	0.055	0.033	0.033	0.027	0.038

FDR, false discovery rate.

### Moderated mediation analysis

**Fig. 2.**
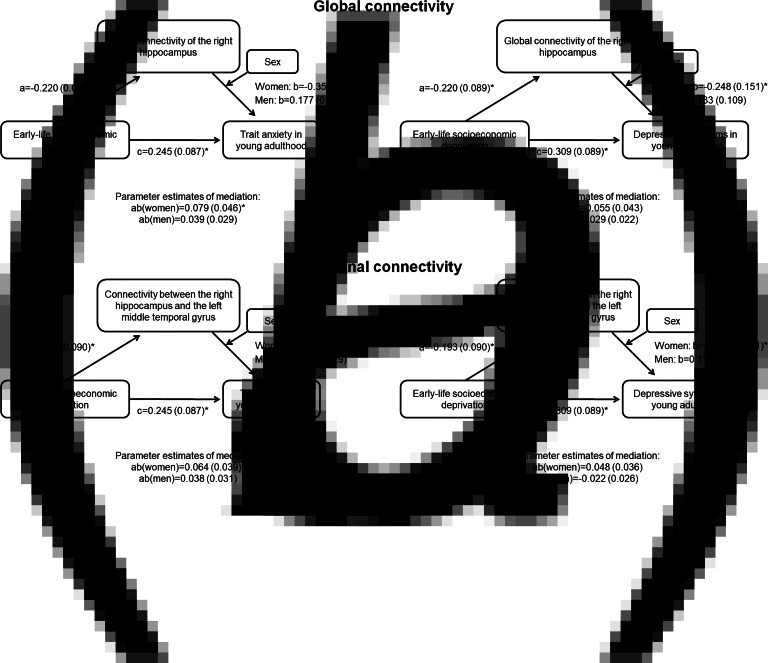
Moderated mediation analysis. Results are *B* (standard error); *p < 0.05. (*a*) Global connectivity of the right hippocampus as a mediator between early-life socioeconomic deprivation and mental health in young adulthood, conditional on sex. (*b*) Connectivity between the right hippocampus and the left middle temporal gyrus as a mediator between early-life socioeconomic deprivation and mental health in young adulthood, conditional on sex.

Global connectivity of the right hippocampus as well as the connectivity between the right hippocampus to the left MTG fulfilled the criteria to be assessed as mediators. Global connectivity of the right hippocampus mediated the relationship between ELSD and trait anxiety in women (Fig. 2*a*; indirect effect *B* = 0.08; 95% CI 0.01–0.19), but not men (indirect effect *B* = −0.04; 95% CI −0.11 to 0.01). In line with this, the connectivity between the right hippocampus and the left MTG mediated the relationship between ELSD and trait anxiety in women (Fig. 2*b*). The moderated mediation did not reach significance in either sex for depressive symptoms as outcome.

## Discussion

We aimed to determine whether ELSD predicts differences in mental health in young adulthood and find potential structural and functional biomarkers of such associations. We demonstrated that socioeconomic deprivation experienced during the first 18 months of life is associated with depressive symptoms and trait anxiety in both men and women. Underlying biomarkers seem to be sex-specific; we found that lower connectivity of the right hippocampus, in particular connectivity to the left MTG, may mediate the association of ELSD with trait anxiety in women. Biomarkers underlying the association with depressive symptoms and the role of the hippocampal volume are less clear.

This study is important in the context of identifying the roots of inequalities in the mental health of young adults. Even though we cannot establish causal relationships based on observational design, this study builds on previous animal experiments, which demonstrated functional and structural changes of the brain in subjects who were exposed to a less resourceful and stressful environment in early life (Chaloner & Greenwood-Van Meerveld, 2013; McCormick, Smythe, Sharma, & Meaney, 1995; Spasojevic et al., 2012). Our findings are in line with epidemiological studies that suggest a role of early-life socioeconomic adversities on the emergence of depressive symptoms and anxiety during the life-course (Angelini, Howdon, & Mierau, 2018; Barch et al., 2016; Najman et al., 2010; Rao et al., 2010; Spence, Najman, Bor, O'Callaghan, & Williams, 2002).

Previous studies operationalized early-life socioeconomic resources in multiple ways, ranging from income-to-needs ratio, crowding, parental education to a number of household utilities (Cermakova et al., 2018; Galobardes, Shaw, Lawlor, Lynch, & Davey Smith, 2006a, 2006b). We demonstrated that specifically subjective socioeconomic deprivation reported by mothers predicts poor mental health of the offspring two decades later. These findings held after accounting for a number of objective markers of SEP, such as education of parents or income, and also for mother's mental health. Previous studies suggested that subjective assessment of one's position in the social hierarchy is a more powerful predictor of subsequent morbidity than objective measures (Singh-Manoux, Marmot, & Adler, 2005). The underlying mechanism behind a socioeconomic gradient in health has been proposed to lie in the brain, in particular in the chronic stress that accompanies a lack of control over life and inability to be a fully recognized member of the society (Marmot, 2004). Here we expand these findings suggesting that the gradient in socioeconomic deprivation reported by the mothers is reflected even in the brain of their offspring.

The variable on ELSD, which captures the inability of the household to pay for basic needs, relates in particular to personal socioeconomic deprivation. This variable is reflective of the socio-political context of young families living in a CEE country, which was going through an economic transition. It measures subjectively viewed deprivation experienced by the sample closely after the fall of the Berlin wall, when inequalities in objective socioeconomic indicators, such as income, were small, and did not largely affect access to social or health care services. However, this measure reflects socioeconomic deprivation in other contexts as well. In the Avon Longitudinal Study of Parents and Children in the UK, it was the strongest predictor of mental health problems in childhood (Russell, Ford, & Russell, 2015). A similarly constructed variable is used also in studies of older adults, such as the Whitehall II Study in the UK (Chandola, Bartley, Sacker, Jenkinson, & Marmot, 2003). Furthermore, a similar measure is a strong predictor of health, including mental health in adults living in CEE after the post-communist socioeconomic transition (Bobak, Pikhart, Rose, Hertzman, & Marmot, 2000; Cermakova, Pikhart, Kubinova, & Bobak, 2020a; Cermakova, Pikhart, Ruiz, Kubinova, & Bobak, 2020b). Its association to depressive symptoms was found consistently present in both men and women living in CEE, but relative to the Czech Republic, the magnitude of the association was slightly higher in Poland and lower in Russia (Bobak et al., 2006).

Socioeconomic disadvantages in early-life have been consistently associated with alterations in the hippocampus, in particular its lower volume (Staff et al., 2012), which was, in turn, proposed to be a core biomarker of anxiety and depression (Gorka et al., 2014; Koolschijn, van IJzendoorn, Bakermans-Kranenburg, & Crone, 2013; Rao et al., 2010; Videbech & Ravnkilde, 2004). Two theories emerged, one suggesting that stress-related hypercortisolemia leads to hippocampal atrophy (McEwen, 2000), and the second one proposing that inherited lower hippocampal volume increases susceptibility to stress and therefore development of mental disorders (Lyons, Yang, Sawyer-Glover, Moseley, & Schatzberg, 2001). There are indications of a stronger role of the right hippocampal volume in the emergence of anxiety and depression (Pruessner et al., 2008; Qiu et al., 2013; Videbech & Ravnkilde, 2004). We found that worse ELSD correlated with a lower volume of the right, not left, hippocampus only in men; however, the volume of the right hippocampus was not related to mental health. Surprisingly, there were indications that higher volume of the left hippocampus may be associated with more depressive symptoms and trait anxiety in both sexes (even though the associations did not pass corrections for multiple testing). Hippocampal volumes were not related to functional connectivity (data not presented).

There may be several interpretations of these unexpected findings. Smaller hippocampus may not necessarily imply its worse function. Despite evidence that lower hippocampal volume is associated with clinical symptoms (Arnone et al., 2013), in non-clinical samples and especially young individuals, results commonly vary from finding none (Hayakawa et al., 2013; McLaren et al., 2016; Rusch, Abercrombie, Oakes, Schaefer, & Davidson, 2001) or even a positive relationship between higher hippocampal volume and mental health symptoms (Besteher et al., 2019). Hippocampal volume may be reduced only after repeated periods of mental disorders (Videbech & Ravnkilde, 2004). Therefore, in this young non-clinical sample, our results could indicate an increased use of hippocampus in individuals facing subclinical depression and anxiety or a resilience effect in a form of compensation of hippocampal volume, which would prevent them from transitioning to poorer mental health.

On the contrary, alteration in hippocampal connectivity may be a more sensitive and earlier biomarker of the association of ELSD to mental health. Recently, Barch et al. found that early-life poverty, assessed with income-to-needs ratio, predicted resting-state connectivity of bilateral hippocampus in children (Barch et al., 2016). Specifically, the connectivity between the left hippocampus and the right superior frontal gyrus mediated the relationship between early-life poverty and negative mood (Barch et al., 2016). Even though we cannot exclude that we lack power to detect associations, our study does not suggest that hippocampal connectivity is a biomarker underlying the association of ELSD with depressive symptoms. Contrary to Barch et al. (2016), we suggest that the connectivity of the right hippocampus, not the left one, may be a biomarker of exposure to ELSD, manifesting with trait anxiety.

Right-left asymmetry of the hippocampus in anxiety has been described on an anatomical, functional and neurochemical level (Spasojevic et al., 2012; Zhang, Chen, Liu, & Feng, 2020). Findings point towards the right hemisphere-dominant behaviour associated with fear and anxiety (Sakaguchi & Sakurai, 2017). Zhang et al. found that lower volume of the right hippocampus as well as lower connectivity between the right hippocampus and the right inferior parietal lobule correlated with stronger trait anxiety (Zhang et al., 2020). Others observed right-left asymmetry of norepinephrine in the hippocampus in animals exposed to strong stress (Spasojevic et al., 2012). Therefore, we conclude that the right hippocampus and its connectivity to other brain regions may play a major role in the pathogenesis of trait anxiety.

We present a novel finding that decreased connectivity between the right hippocampus to the left MTG may mediate the association of ELSD with trait anxiety in women. The role of the MTG is well documented in anxiety disorders: lower functional activity in the MTG was observed in patients with generalized anxiety disorders (Qiao et al., 2017). The left MTG was suggested to be a network hub for resting-state functional connectivity in social anxiety disorder (Liu et al., 2015; Yun et al., 2017). In line with this, Liao et al. found increased functional connectivity of the right parahippocampal/hippocampal gyrus to the left MTG among people with social anxiety disorder (Liao et al., 2011). It was proposed that the MTG has a role in inhibiting the excessive corticolimbic activity in anxiety disorders and mediating fear and avoidance behaviour (Heeren & McNally, 2016; Liao et al., 2011).

As we found that ELSD was associated more strongly with depressive symptoms in women than in men and the connectivity of the right hippocampus mediated the association between ELSD and trait anxiety only in women, we propose that ELSD may be mirrored more strongly in the brain function of women when compared to men. This is in line with previous studies that suggested women to be more sensitive to the effects of early-life adversities (Burghy et al., 2012; Javanbakht et al., 2016; Murgatroyd & Spengler, 2011; Weinstock, 2007). Evidence from animal experiments demonstrated that female rats exhibited greater nociceptive responses (Chaloner & Greenwood-Van Meerveld, 2013) and higher hypothalamic-pituitary-adrenal (HPA) axis reactivity (McCormick et al., 1995) following early-life stress, when compared to males. In studies on humans, women showed stronger effects of poverty on their implicit emotional reactivity (Javanbakht et al., 2016), a greater neuroendocrine reaction to early-life stress (Weinstock, 2007) and more robust epigenetic changes in response to early social environmental factors (Murgatroyd & Spengler, 2011). In women, but not men, greater early-life stress predicted increased childhood cortisol levels, which, in turn, was associated with decreased functional connectivity of the brain 14 years later (Burghy et al., 2012).

Disruption of the HPA axis regulation is a basis for anxiety and studies suggest sex-specific neurobiological mechanisms behind the role of hippocampus in the regulation of HPA axis and subsequent anxiety symptoms (Marques et al., 2016). Taking all this literature into account, HPA axis reactivity may be a key biological mechanism underlying our findings. The decreased connectivity of the right hippocampus with regions involved in emotion processing may indicate reduced HPA axis regulation in women as a response to ELSD, manifesting in trait anxiety. Further research should address biomarkers underlying the association of ELSD to depressive symptoms in both sexes, given that trait anxiety often precedes the development of depression (Parker et al., 1999).

Several limitations need to be mentioned. Most of our participants have highly educated parents and the socioeconomic deprivation score in our sample was rather low. This likely leads to underestimation of the associations we found. The effect of socioeconomic deprivation in different life stages was not taken into account as these data in adolescence were collected only for a small sub-sample of individuals and they were not collected in young adulthood. In spite of the limitations, these findings indicate that early preventive strategies targeted at children from socioeconomically deprived families may yield long-lasting benefits for their mental health.

## Supplementary material

For supplementary material accompanying this paper visit https://doi.org/10.1017/S0033291720004754. click here to view supplementary material
