# Supplementary material for: Socioeconomic deprivation in early life and symptoms of depression and anxiety in young adulthood: mediating role of hippocampal connectivity
Source: Psychol Med. 2020 Dec 17;52(13):2671–80. doi: 10.1017/S0033291720004754 (PMC9647532; doi:10.1017/S0033291720004754)
Supplement: Supplementary file 1 [file S0033291720004754sup001.docx]

**SUPPLEMENT**

**Content:**

1. Supplementary methods
   1. Participants
   2. Early-life socioeconomic deprivation
   3. Covariates
   4. Analysis of hippocampal volume
   5. Analysis of functional connectivity
   6. Multiple testing strategies
2. Supplementary tables
   1. Table S1 Bivariate correlations between main measures
   2. Table S2 Association of early-life covariates with mental health
   3. Table S3 Correlation between early-life socioeconomic deprivation and right hippocampal subfields in men
   4. Table S4 Bivariate correlations between right hippocampal subfield volumes
   5. Table S5 Bivariate correlations between volumes of subfield of the right and left hippocampus
   6. Table S6 Cluster of voxels connected with the right hippocampus
   7. Table S7 Cluster of voxels connected with the left hippocampus
3. Supplementary figures
   1. Figure S1 Recruitment flow diagram
   2. Figure S2 Distribution of the final socioeconomic deprivation score in early life
   3. Figure S3 Connectivity between right hippocampus and other regions of interest
4. **SUPPLEMENTARY METHODS**
   1. Participants

Individuals participating in the Biomarkers and Underlying Mechanisms of Vulnerability to Depression (VULDE) neuroimaging follow-up of the European Longitudinal Study of Pregnancy and Childhood (ELSPAC) cohort were previously described (Mareckova *et al.*, 2019a, Mareckova *et al.*, 2019b, Mareckova *et al.*, 2020, Mareckova *et al.*, 2018). The neuroimaging follow-up was conducted on 131 young adults (age 23/24) who were chosen from the large cohort based on first-come, first-serve basis (Supplementary Figure S1).

- 1. Early-life socioeconomic deprivation

To create a measure that would sufficiently capture early-life socioeconomic deprivation (ELSD), we combined the socioeconomic deprivation score at 6 and 18 months of the offspring. At both time points, mothers answered how difficult it is to secure food, clothes, heating, rent/other fees, and things necessary for the child. The 4 possible answers ranged from “very difficult” (coded as 3) to “not difficult at all” (coded as 0). Scores on these five items were summed-up, creating a socioeconomic deprivation score reaching from 0 to 15 (higher values indicating more severe deprivation). The measure of socioeconomic deprivation was used in previous epidemiological studies and is a strong predictor of health, including mental health, in Central and Eastern Europe (CEE) after the post-communist socioeconomic transition (Bobak *et al.*, 2000, Cermakova *et al.*, 2020a, Cermakova *et al.*, 2020b). The region of CEE is characterized by an especially high burden of mental disorders and treatment gap, has been previously largely underrepresented in studies on mental health and is currently undergoing a reform of mental health care (Kagstrom *et al.*, 2019, Winkler *et al.*, 2018).

The socioeconomic deprivation score at 6 months of the offspring reached the median of 2 with interquartile range (IQR) 4 (mean 3.1, range 0-12) and showed a good internal consistency (Cronbach´s alpha 0.81). The score at 18 months reached the median of 2 (IQR 4, mean 3.1, range 0-11) and had a good internal consistency as well (Cronbach´s alpha 0.82). The scores at months 6 and 18 were positively correlated (Spearmans´s rho=0.38; p<0.001). The final socioeconomic deprivation score was calculated as the mean of the scores for 6 and 18 months (median 2.5, IQR 3.5; mean 3.1; range 0-10). Supplementary Figure S2 presents distribution of the final score. It was entered into the analyses as a z-score.

- 1. Covariates

We took into account early-life characteristics that could act as confounding factors in the association of ELSD with mental health in young adulthood. Age was not used as a covariate for adjustment as there was no relationship with neither ELSD (Spearman´s rho -0.03; p=0.73) nor with any studied outcome (depressive symptoms: Spearman´s rho -0.11, p=0.22; trait anxiety: Spearman´s rho -0.16, p=0.08; global connectivity of the right hippocampus: Spearman´s rho -0.04, p=0.66; volume of the right hippocampus: Spearman´s rho -0.05, p=0.58; volume of the left hippocampus: Spearman´s rho -0.091, p=0.32). Covariates related to early-life socioeconomic position (SEP) were identified based on literature (Cermakova *et al.*, 2018, Galobardes *et al.*, 2006a, b) as seven indicators: 1) mother´s education, 2) father´s education, 3) father´s occupation, 4) household income 5) basic utilities, 6) household items and 7) crowding ratio. This data was collected from questionnaires administered to the mother and her partner at 4 different times: upon enrollment to the study, at 6 months, at 18 months and at 3 years of the child´s life. Education of both parents was coded in 8 different categories: 1) primary, 2) vocational without high school graduation, 3) vocational with high school graduation, 4) specialized high school with graduation, 5) general high school with graduation, 6) post-high school graduation study, 7) university education and 8) postgraduate education.

As maternity leave in the Czech Republic usually lasts for 3 years, we considered only the occupation of the father, coded in the International standard Classification of Occupations (ISCO) 1988 categories. Using the previously suggested algorithm (Ganzeboom and Treiman, 1996), we recoded the ISCO 88 categories into 10 classes according to the Erikson, Goldthorpe and Portocareros (EGP) scheme (Erikson *et al.*, 1979). The variable “household income” is the sum of netto incomes of the mother and her partner, side incomes as well as social benefits.

The variable “basic utilities” is the sum of the following 9 utilities that the participants have at home for their own use: kitchen, flush toilet, running hot water, bathtub, shower, garden/yard, balcony/terrace/loggia, phone and car. The variable “household items” is the sum of owning the following 10 items: fridge, washing machine, mangle, dishwasher, freezer, microwave, vacuum cleaner, Remoska/grill/frying pot, sewing machine and kitchen robot. “Crowding ratio” was calculated by dividing the number of household members by the number of rooms in the household.

While the questionnaires filled-in by the mother included both questions about her own as well as her partner´s education and occupation, the questionnaires filled-in by the partner concerned only his own. In case of missing data in the partner´s questionnaire, we used the partner´s education and occupation reported by the mother. We consider information about the partner as identical to the father. All other data was included in the questionnaires administered to the mother. For items, on which data was available at different time points during the three years, we calculated the arithmetic average from them. We re-coded each indicator of early-life SEP so that higher values indicate more adverse conditions.

The measure of ELSD correlates weakly with basic utilities (Spearman´s rho=0.19; p=0.04) and household items (Spearman´s rho=0.20; p=0.05) but not with other measures (Supplementary Table S1), suggesting that each of these covariates captures different aspects of early-life SEP that could confound the association of ELSD with outcomes in young adulthood.

Covariates related to mother´s mental health were mother´s depression and mother´s anxiety (Mareckova *et al.*, 2020, Mareckova *et al.*, 2018). Mother´s depression was measured at the 20th week of gestation using a self-reported questionnaire. This questionnaire included the following nine questions answered on a 4-point Likert scale: 1) Were you looking forward to future events? 2) Did you blame yourself unnecessarily when something did not work out? 3) Did you worry or feel anxious without a relevant reason? 4) Were you afraid or did you panic without a relevant reason? 5) Were you overwhelmed by everything in your life? 6) I was so unhappy I had sleep difficulties. 7) I was sad and miserable. 8) I was so unhappy that I had to cry. 9) I was thinking about hurting myself. Details about this measure can be found elsewhere (Mareckova *et al.*, 2020).

Mother´s anxiety was assessed during the first weeks after birth with a questionnaire, which included 36 questions on anxious behavior and co-dependence such as being worried about other people’s opinions, changing behavior in order to please others, being insecure when meeting new people, being worried to say what she thinks because others might not like her, being worried to be criticized, being anxious when saying good bye, being worried to lose a friend, all answered on a 4-point Likert scale. Details have been previously published (Mareckova *et al.*, 2018).

- 1. Analysis of hippocampal volume

The method for the calculation of the right and left hippocampus was described in our previous study (Mareckova *et al.*, 2018). Briefly, T1-weighted data were processed using Freesurfer version 6.0 and volumes of hippocampal subfields were calculated according to a method by Iglesias (Iglesias *et al.*, 2015). This is an automated analysis based on an atlas of the hippocampal subfields constructed with the use of ultra-high-resolution MRI. The method then utilizes an algorithm based on Bayesian inference. The hippocampal subfields are left and right parasubiculum, presubiculum, subiculum, cornu ammonis (CA) 1, CA2/3, CA4, granule cell layer of the dentate gyrus (GC/DG), hippocampal-amygdaloid transition area (HATA), fimbria, molecular layer, hippocampal fissure and hippocampal tail. Segmentation of the hippocampal subfields was visually inspected and all subjects passed the quality control. The data was extracted from Freesurfer into SPSS (IBM SPSS Statistics, IBM Corp, Armonk, NY) for statistical analysis.

- 1. Analysis of hippocampal connectivity

Functional connectivity analysis was performed using CONN Functional Connectivity Toolbox version 18.b. and its default pre-processing pipeline (Whitfield-Gabrieli and Nieto-Castanon, 2012). First, functional images were realigned, un-warped, and slice-timing corrected (interleaved bottom-up). Next, the images were co-registered with structural data and spatially normalized to the Montreal Neurological Institute (MNI) space.

Several steps have been undertaken to ensure that the hippocampal connectivity is not impacted by head motion. Potential outlier scans due to subjects´ head movement were assessed using the ARtifact Detection Tools (ART) - based scrubbing. The threshold for potential identification of outliers was set at the 95^th^ percentile in normative samples, a conservative setting for ART.

The first-level covariates were:

1. Realignment – it contains the actual subject movement parameters estimated across time, so these are time series representing how the subject moved within the scanner.
2. Quality Assurance time series – they represent two following parameters: First, how the global signal in the brain is changing from one scan to the next. Second, the Framewise Displacement, which is a measure of how much the subject moved from one acquisition to the next. High values represent a high level of motion between two adjacent scans, which is a good indicator of potential outliers.
3. Scrubbing – this is the identification of potential outliers in these two timeseries, a result of ART. It identifies scans that may have been potential outliers because they have either a high level of motion in the subject or a lot of global signal changes.

Finally, the images were smoothed using a Gaussian kernel of 8 mm full width at half maximum (FWHM) and de-noised. De-noising has been proven to improve the quality and replicability of functional connectivity measures. It addresses a number of residual physiological and motion effects that are present even after preprocessing. The method implemented within CONN toolbox is anatomical CompCor. Details about the CompCor method are provided elsewhere (Behzadi *et al.*, 2007). The CompCor method assumes the presence of “noise region of interest (ROI)”, such as white matter, ventricles or large vessels, in which fluctuations in time are unlikely to be caused by neural activity and are primarily reflection of physiological noise. Signal from the noise ROI can be used to model physiological fluctuations in gray matter. This method is extracting a representative noise signal from white matter regions and a representative noise signal from the cerebrospinal fluid and it is removing anything that correlates with those noise components from the BOLD signal for every voxel, with the realignment parameters (subject motion parameters that were estimated during realignment), and also with the scrubbing covariate, so this effectively takes out those outlier scans (“scrubbing” – removing the influence of the effect of those outlier scans that have been deemed to be potential outliers). All of these are removed in a single linear regression step and the residuals of that linear regression are the resulting clean signal, without the effect of all these noise effects.

The advantage of this method (*effectively* removing the outlier scans using dummy-coded regressors during de-noising rather than *actually* removing the outlier scans from the functional volumes) is that it maintains the temporal resolution of the data. On the contrary, by *actually* removing time points, the length of scans is altered, it disrupts the continuity of the time series, which affects processes like band-pass filtering that assume continuous data. Distribution of connectivity values and BOLD time series were visually checked after de-noising, all participants passed the requirements. The data were band-pass filtered to .008 Hz–.09 Hz.

In addition, we also tested whether between-subject differences in head motion during the resting state scan are related to ELSD or mental health in young adulthood. We created a subject-level measure that computes the amount of movement for each subject. Specifically, we computed the average displacement (mean 0.011, SD 0.057) as well as maximum displacement (median 0.298, IQR 0.292). Neither of these parameters correlated with ELSD: average displacement r=0.059; p=0.522 / Spearman´s rho 0.083; p=0.365; maximum displacement r=0.039; p=0.674 / Spearman´s rho 0.085; p=0.350. The parameters did not correlate with depressive symptoms in young adulthood either: average displacement r=-0.057; p=0.532 / Spearman´s rho -0.017; p=0.848; maximum displacement r=0.077; p=0.400 / Spearman´s rho 0.091; p=0.319). They did not correlate with trait anxiety in young adulthood either: average displacement r=-0.037; p=0.682 / Spearman´s rho -0.042; p=0.648; maximum displacement r=0.104; p=0.256 / Spearman´s rho 0.035; p=0.702.

- 1. Multiple testing strategies

In Step 1 (association of ELSD with mental health in young adulthood), we corrected the associations of ELSD with depressive symptoms and trait anxiety for false discovery rate (FDR), using Benjamini-Hochberg method. In Step 2 (analysis of hippocampal volume), the associations of ELSD with the right and left hippocampal volumes, the associations of both hippocampal volumes with mental health outcomes and the association of ELSD with hippocampal subfields were adjusted for FDR, also using Benjamini-Hochberg method. In Step 3 (analysis of hippocampal connectivity), we used the following multiple testing strategy for the analysis of global hippocampal connectivity: seed-to-voxel results are reported when significant at a voxel-wise threshold of level of p<.001 uncorrected and a cluster-level threshold of p<.05 FDR corrected. Furthermore, in the analysis of the regional hippocampal connectivity, a p corrected for FDR <.05 identified statistically significant correlations between the ROIs. In Step 4 (moderated mediation analysis), we did not correct for multiple testing as we only assessed mediators that fulfilled the pre-specified criteria.

**SUPPLEMENTARY TABLES**

**Table S1** Bivariate correlations between main measures

|  | Deprivation | STAI-T | STAI-S | MFQ | Income | Mother´s education | Father´s education | Occupation | Utilities | Items | Crowding |
| --- | --- | --- | --- | --- | --- | --- | --- | --- | --- | --- | --- |
| Deprivation | / | 0.224* | 0.054 | 0.298** | 0.132 | 0.081 | -0.017 | 0.172 | 0.186* | 0.197* | 0.126 |
| STAI-T | 0.224* | / | 0.408** | 0.714** | -0.021 | -0.183 | -0.026 | -0.048 | -0.071 | -0.138 | -0.008 |
| STAI-S | 0.054 | 0.408** | / | 0.412** | 0.028 | -0.050 | 0.000 | -0.038 | -0.010 | -0.095 | 0.107 |
| MFQ | 0.298** | 0.714** | 0.412** | / | 0.016 | -0.021 | -0.001 | -0.023 | 0.096 | -0.114 | 0.151 |
| Income | 0.132 | -0.021 | 0.028 | 0.016 | / | 0.195 | 0.331** | 0.345** | 0.361** | 0.219* | 0.056 |
| Mother´s education | 0.081 | -0.183 | -0.050 | -0.021 | 0.195 | / | 0.295** | 0.430** | 0.235* | -0.008 | 0.139 |
| Father´s education | -0.017 | -0.026 | 0.000 | -0.001 | 0.331** | 0.295** | / | 0.756** | 0.114 | 0.029 | 0.092 |
| Occupation | 0.172 | -0.048 | -0.038 | -0.023 | 0.345** | 0.430** | 0.756** | / | 0.217* | 0.187 | 0.084 |
| Utilities | 0.186* | -0.071 | -0.010 | 0.096 | 0.361** | 0.235* | 0.114 | 0.217* | / | 0.097 | 0.461** |
| Items | 0.197 | -0.138 | -0.095 | -0.114 | 0.219* | -0.008 | 0.029 | 0.187 | 0.097 | / | 0.228** |
| Crowding | 0.126 | -0.008 | 0.107 | 0.151 | 0.056 | 0.139 | 0.092 | 0.084 | 0.461** | 0.228** | / |

Data are Spearman´s rho correlations; *p<0.05, **p<0.001

Deprivation=socioeconomic deprivation in early-life, STAI-T=trait anxiety; STAI-S=state anxiety; MFQ= depressive symptoms assessed by Mood and Feelings Questionnaire; income=household income; utilities=basic utilities; items=household items; crowding=crowding ratio

**Table S2** Association of early-life covariates with mental health

|  | Depressive symptoms | | Trait anxiety | |
| --- | --- | --- | --- | --- |
|  | Unadjusted | Adjusted | Unadjusted | Adjusted |
| Covariates related to early-life socioeconomic position |  |  |  |  |
| Household income | 0.06 (-0.08; 0.19) | 0.07 (-0.08; 0.22) | 0.01 (-0.05; 0.07) | 0.02 (-0.05; 0.08) |
| Mother´s education | -0.03 (-0.18; 0.12) | -0.08 (-0.24; 0.08) | -0.06 (-0.13; 0.00) | -0.08 (-0.15; -0.01)* |
| Father´s education | -0.02 (-0.17; 0.13) | -0.15 (-0.38; 0.08) | -0.01 (-0.07; 0.06) | -0.04 (-0.14; 0.06) |
| Father´s occupation | 0.03 (-0.10; 0.16) | 0.21 (-0.05; 0.47) | 0.01 (-0.04; 0.07) | 0.08 (-0.03; 0.19) |
| Basic utilities | 0.04 (-0.09; 0.18) | -0.14 (-0.33; 0.05) | -0.03 (-0.09; 0.03) | -0.07 (-0.16; 0.01) |
| Household items | -0.10 (-0.24; 0.05) | -0.21 (-0.37; -0.06)* | -0.05 (-0.12; 0.01) | -0.08 (-0.15; -0.01)* |
| Crowding ratio | 0.12 (-0.01; 0.25) | 0.17 (-0.00; 0.35) | -0.00 (-0.06; 0.06) | 0.05 (-0.03; 0.13) |
| Covariates related to mother´s mental health |  |  |  |  |
| Mother´s depression | 0.02 (-0.02; 0.05) | 0.02 (-0.02; 0.05) | 0.01 (-0.01; 0.02) | 0.01 (-0.01; 0.02) |
| Mother´s anxiety | -0.11 (-0.46; 0.24) | -0.04 (-0.43; 0.34) | 0.01 (-0.14; 0.16) | -0.00 (-0.17; 0.17) |

Results are B with 95% confidence intervals for the association of early-life covariates with mental health outcomes derived from linear regression.

*p<0.05

Adjusted results show the association of early-life covariates with a mental health outcome, including variables either in Model 1 (covariates related to early-life socioeconomic position) or Model 2 (covariates related to mother´s mental health). Covariates related to early-life socioeconomic position were entered into the model as follows: early-life socioeconomic deprivation, sex, household income, mother´s education, father´s education, father´s occupation, basic utilities, household items and crowding ratio. Covariates related to mother´s mental health were entered into the model as follows: early-life socioeconomic deprivation, sex, mother´s depression and mother´s anxiety.

**Table S3** Correlation between early-life socioeconomic deprivation and right hippocampal subfields in men

|  | Men | |
| --- | --- | --- |
|  | r | p value /  p-FDR |
| Subfields of the right hippocampus |  |  |
| Subiculum | -0.038 | 0.775/0.845 |
| Presubiculum | -0.087 | 0.511/0.681 |
| Parasubiculum | 0.030 | 0.822/0.822 |
| CA1 | -0.342 | 0.008/0.048 |
| CA3 | -0.302 | 0.020/0.080 |
| CA4 | -0.273 | 0.037/0.074 |
| GC/DG | -0.277 | 0.034/0.082 |
| HATA | -0.179 | 0.176/0.302 |
| Fimbria | 0.056 | 0.673/0.808 |
| Molecular layer | -0.293 | 0.024/0.072 |
| Hippocampal fissure | -0.365 | 0.005/0.060 |
| Hippocampal tail | -0.151 | 0.255/0.383 |

CA, cornu ammonis; GC/DG, granule cell layer of the dentate gyrus; HATA, hippocampal-amygdaloid transition area; p-FDR, p value corrected for false discovery rate

**Table S4** Bivariate correlations between volumes of subfield of the right hippocampus

|  | Subiculum | Pre-subiculum | Para-subiculum | CA1 | CA3 | CA4 | GC/  DG | HATA | Fimbria | Molecular layer | Fissure | Tail | Total |
| --- | --- | --- | --- | --- | --- | --- | --- | --- | --- | --- | --- | --- | --- |
| Subiculum | / | 0.74** | 0.31* | 0.59** | 0.21* | 0.58** | 0.62** | 0.27* | 0.36** | 0.75** | 0.31* | 0.31* | 0.80** |
| Pre-subiculum | 0.74** | / | 0.52** | 0.37** | -0.02 | 0.36** | 0.39** | 0.14 | 0.32** | 0.52** | 0.27* | 0.28* | 0.62** |
| Para-subiculum | 0.31** | 0.52** | / | 0.04 | -0.09 | 0.11 | 0.08 | 0.18 | 0.26* | 0.14 | 0.03 | 0.19* | 0.26* |
| CA1 | 0.59** | 0.37** | 0.04 | / | 0.51** | 0.72** | 0.78** | 0.42** | 0.30* | 0.90** | 0.32** | 0.20* | 0.85** |
| CA3 | 0.21* | -0.02 | -0.09 | 0.51** | / | 0.79** | 0.75** | 0.21* | -0.08 | 0.64** | 0.21* | 0.03 | 0.56** |
| CA4 | 0.58** | 0.36** | 0.11 | 0.72** | 0.79** | / | 0.98** | 0.25* | 0.07 | 0.88** | 0.22* | 0.19* | 0.85** |
| GC/DG | 0.62** | 0.39** | 0.08 | 0.78** | 0.75** | 0.98** | / | 0.31** | 0.15 | 0.91** | 0.24** | 0.18* | 0.88** |
| HATA | 0.27* | 0.14 | 0.18 | 0.42** | 0.21* | 0.25* | 0.31** | / | 0.51** | 0.33** | 0.08 | -0.05 | 0.37** |
| Fimbria | 0.36** | 0.32** | 0.26* | 0.30* | -0.08 | 0.07 | 0.15 | 0.51** | / | 0.25* | -0.03 | -0.05 | 0.31* |
| Molecular layer | 0.75** | 0.52** | 0.14 | 0.90** | 0.64** | 0.88** | 0.91** | 0.33** | 0.25* | / | 0.31* | 0.24* | 0.96** |
| Fissure | 0.31* | 0.27* | 0.03 | 0.32** | 0.21* | 0.22* | 0.24* | 0.08 | -0.03 | 0.31* | / | 0.10 | 0.34** |
| Tail | 0.31* | 0.28* | 0.19* | 0.20* | 0.03 | 0.19* | 0.18* | -0.05 | -0.05 | 0.24** | 0.10 | / | 0.43** |
| Total | 0.80** | 0.62** | 0.26* | 0.85** | 0.56** | 0.85** | 0.88** | 0.37** | 0.31* | 0.96** | 0.34** | 0.43** | / |

CA, cornu ammonis; GC/DG, granule cell layer of the dentate gyrus; HATA, hippocampal-amygdaloid transition area

Fissure=hippocampal fissure; tail=hippocampal tail; total=total volume of the right hippocampus

*p<0.05; **p<0.001

**Table S5** Bivariate correlations between volumes of subfield of the right and left hippocampus

|  |  | Left hippocampus | | | | | | | | | | | | |
| --- | --- | --- | --- | --- | --- | --- | --- | --- | --- | --- | --- | --- | --- | --- |
|  |  | Subiculum | Pre-subiculum | Para-subiculum | CA1 | CA3 | CA4 | GC/  DG | HATA | Fimbria | Molecular layer | Fissure | Tail | Total |
| Right hippocampus | Subiculum | 0.75** | 0.55** | 0.25* | 0.51** | 0.16 | 0.46** | 0.49** | 0.27* | 0.29* | 0.60** | 0.26* | 0.30* | 0.65** |
|  | Pre-subiculum | 0.60** | 0.68** | 0.38** | 0.41** | 0.06 | 0.39** | 0.42** | 0.17 | 0.19* | 0.49** | 0.13 | 0.26* | 0.57** |
|  | Para-subiculum | 0.24* | 0.29* | 0.62** | 0.08 | -0.21* | -0.03 | 0.01 | 0.05 | 0.16 | 0.06 | -0.07 | 0.10 | 0.14 |
|  | CA1 | 0.53** | 0.25** | 0.07 | 0.60** | 0.34** | 0.49** | 0.53** | 0.37** | 0.32** | 0.57** | 0.25* | 0.28* | 0.59** |
|  | CA3 | 0.19* | 0.01 | 0.03 | 0.36** | 0.48** | 0.38** | 0.37** | 0.10 | 0.08 | 0.36** | 0.21* | 0.18* | 0.34** |
|  | CA4 | 0.52** | 0.32** | 0.20* | 0.55** | 0.38** | 0.55** | 0.56** | 0.25* | 0.22* | 0.59** | 0.19* | 0.30* | 0.62** |
|  | GC/DG | 0.54** | 0.33** | 0.16 | 0.59** | 0.39** | 0.56** | 0.60** | 0.29* | 0.26* | 0.62** | 0.22* | 0.29* | 0.64** |
|  | HATA | 0.17 | 0.18* | 0.14 | 0.33** | 0.28* | 0.26* | 0.33** | 0.58** | 0.48** | 0.25* | 0.02 | -0.11 | 0.26* |
|  | Fimbria | 0.31** | 0.26* | 0.17 | 0.29* | -0.16 | 0.15 | 0.23* | 0.45** | 0.62** | 0.24* | 0.04 | 0.03 | 0.32** |
|  | Molecular layer | 0.63** | 0.40** | 0.17 | 0.63** | 0.37** | 0.55** | 0.58** | 0.31* | 0.32** | 0.66** | 0.27* | 0.33** | 0.68** |
|  | Fissure | 0.30* | 0.22* | 0.16 | 0.30* | 0.18 | 0.27* | 0.24* | 0.01 | -0.06 | 0.30* | 0.52** | 0.10 | 0.30* |
|  | Tail | 0.30* | 0.22* | 0.14 | 0.15 | 0.12 | 0.21* | 0.20* | 0.02 | -0.13 | 0.24* | 0.13 | 0.69** | 0.37** |
|  | Total | 0.68** | 0.47** | 0.26* | 0.64** | 0.36** | 0.59** | 0.61** | 0.34** | 0.31** | 0.69** | 0.26* | 0.44** | 0.75** |

CA, cornu ammonis; GC/DG, granule cell layer of the dentate gyrus; HATA, hippocampal-amygdaloid transition area

Fissure=hippocampal fissure; tail=hippocampal tail; total=total volume of the right hippocampus

*p<0.05; **p<0.001

**Table S6** Cluster of voxels connected to right hippocampus

Coordinates +24; -16; -18

Size: 120 934

5031 voxels covering 62% of atlas.FP r (Frontal Pole Right)

4950 voxels covering 71% of atlas.FP l (Frontal Pole Left)

3152 voxels covering 76% of atlas.Brain-Stem

2606 voxels covering 46% of atlas.Precuneous (Precuneous Cortex)

2421 voxels covering 49% of atlas.sLOC l (Lateral Occipital Cortex, superior division Left)

2274 voxels covering 95% of atlas.TP r (Temporal Pole Right)

2235 voxels covering 94% of atlas.TP l (Temporal Pole Left)

1957 voxels covering 69% of atlas.SFG l (Superior Frontal Gyrus Left)

1827 voxels covering 68% of atlas.SFG r (Superior Frontal Gyrus Right)

1739 voxels covering 59% of atlas.MidFG l (Middle Frontal Gyrus Left)

1711 voxels covering 36% of atlas.sLOC r (Lateral Occipital Cortex, superior division Right)

1707 voxels covering 62% of atlas.MidFG r (Middle Frontal Gyrus Right)

1679 voxels covering 97% of atlas.LG r (Lingual Gyrus Right)

1453 voxels covering 96% of atlas.LG l (Lingual Gyrus Left)

1336 voxels covering 56% of atlas.PC (Cingulate Gyrus, posterior division)

1314 voxels covering 64% of atlas.iLOC r (Lateral Occipital Cortex, inferior division Right)

1306 voxels covering 84% of atlas.Cereb6 r (Cerebelum 6 Right)

1092 voxels covering 53% of atlas.iLOC l (Lateral Occipital Cortex, inferior division Left)

1062 voxels covering 78% of atlas.PaCiG r (Paracingulate Gyrus Right)

1046 voxels covering 81% of atlas.Cereb6 l (Cerebelum 6 Left)

1041 voxels covering 77% of atlas.IC r (Insular Cortex Right)

1032 voxels covering 84% of atlas.pSMG r (Supramarginal Gyrus, posterior division Right)

966 voxels covering 66% of atlas.SPL l (Superior Parietal Lobule Left)

939 voxels covering 70% of atlas.IC l (Insular Cortex Left)

883 voxels covering 83% of atlas.pSMG l (Supramarginal Gyrus, posterior division Left)

878 voxels covering 98% of atlas.Cereb45 l (Cerebelum 4 5 Left)

874 voxels covering 78% of atlas.SubCalC (Subcallosal Cortex)

867 voxels covering 88% of atlas.MedFC (Frontal Medial Cortex)

866 voxels covering 59% of atlas.AG r (Angular Gyrus Right)

861 voxels covering 88% of atlas.CO l (Central Opercular Cortex Left)

854 voxels covering 97% of atlas.CO r (Central Opercular Cortex Right)

826 voxels covering 63% of atlas.PaCiG l (Paracingulate Gyrus Left)

821 voxels covering 92% of atlas.OFusG r (Occipital Fusiform Gyrus Right)

813 voxels covering 100% of atlas.TOFusC r (Temporal Occipital Fusiform Cortex Right)

785 voxels covering 53% of atlas.SPL r (Superior Parietal Lobule Right)

770 voxels covering 29% of atlas.OP l (Occipital Pole Left)

766 voxels covering 30% of atlas.AC (Cingulate Gyrus, anterior division)

761 voxels covering 100% of atlas.Hippocampus l

730 voxels covering 43% of atlas.FOrb l (Frontal Orbital Cortex Left)

727 voxels covering 50% of atlas.FOrb r (Frontal Orbital Cortex Right)

710 voxels covering 76% of atlas.OFusG l (Occipital Fusiform Gyrus Left)

698 voxels covering 100% of atlas.Hippocampus r

681 voxels covering 86% of atlas.Cereb9 r (Cerebelum 9 Right)

670 voxels covering 78% of atlas.Cereb9 l (Cerebelum 9 Left)

669 voxels covering 49% of atlas.pMTG r (Middle Temporal Gyrus, posterior division Right)

652 voxels covering 100% of atlas.TOFusC l (Temporal Occipital Fusiform Cortex Left)

644 voxels covering 98% of atlas.aPaHC r (Parahippocampal Gyrus, anterior division Right)

634 voxels covering 67% of atlas.AG l (Angular Gyrus Left)

626 voxels covering 73% of atlas.pTFusC l (Temporal Fusiform Cortex, posterior division Left)

624 voxels covering 100% of atlas.Ver45 (Vermis 4 5)

616 voxels covering 14% of atlas.PreCG l (Precentral Gyrus Left)

602 voxels covering 24% of atlas.Cereb1 r (Cerebelum Crus1 Right)

595 voxels covering 97% of atlas.Cereb45 r (Cerebelum 4 5 Right)

593 voxels covering 43% of atlas.pMTG l (Middle Temporal Gyrus, posterior division Left)

591 voxels covering 14% of atlas.PreCG r (Precentral Gyrus Right)

580 voxels covering 16% of atlas.PostCG l (Postcentral Gyrus Left)

577 voxels covering 42% of atlas.Thalamus l

569 voxels covering 98% of atlas.aPaHC l (Parahippocampal Gyrus, anterior division Left)

560 voxels covering 44% of atlas.Thalamus r

555 voxels covering 58% of atlas.aSMG l (Supramarginal Gyrus, anterior division Left)

551 voxels covering 86% of atlas.ICC l (Intracalcarine Cortex Left)

535 voxels covering 95% of atlas.PT l (Planum Temporale Left)

518 voxels covering 72% of atlas.pTFusC r (Temporal Fusiform Cortex, posterior division Right)

511 voxels covering 64% of atlas.Putamen r

500 voxels covering 58% of atlas.Putamen l

497 voxels covering 66% of atlas.ICC r (Intracalcarine Cortex Right)

480 voxels covering 19% of atlas.OP r (Occipital Pole Right)

449 voxels covering 56% of atlas.aSMG r (Supramarginal Gyrus, anterior division Right)

441 voxels covering 14% of atlas.PostCG r (Postcentral Gyrus Right)

436 voxels covering 97% of atlas.aMTG l (Middle Temporal Gyrus, anterior division Left)

416 voxels covering 100% of atlas.pSTG r (Superior Temporal Gyrus, posterior division Right)

404 voxels covering 99% of atlas.aMTG r (Middle Temporal Gyrus, anterior division Right)

403 voxels covering 92% of atlas.PT r (Planum Temporale Right)

390 voxels covering 100% of atlas.pPaHC l (Parahippocampal Gyrus, posterior division Left)

387 voxels covering 99% of atlas.pSTG l (Superior Temporal Gyrus, posterior division Left)

377 voxels covering 100% of atlas.PP r (Planum Polare Right)

358 voxels covering 100% of atlas.PP l (Planum Polare Left)

342 voxels covering 100% of atlas.Amygdala r

340 voxels covering 15% of atlas.Cereb1 l (Cerebelum Crus1 Left)

334 voxels covering 62% of atlas.PO r (Parietal Operculum Cortex Right)

334 voxels covering 59% of atlas.PO l (Parietal Operculum Cortex Left)

334 voxels covering 100% of atlas.Ver6 (Vermis 6)

327 voxels covering 100% of atlas.Amygdala l

320 voxels covering 45% of atlas.SMA r (Juxtapositional Lobule Cortex -formerly Supplementary Motor Cortex- Right)

319 voxels covering 100% of atlas.pPaHC r (Parahippocampal Gyrus, posterior division Right)

309 voxels covering 100% of atlas.HG l (Heschl's Gyrus Left)

296 voxels covering 93% of atlas.aTFusC l (Temporal Fusiform Cortex, anterior division Left)

288 voxels covering 99% of atlas.aTFusC r (Temporal Fusiform Cortex, anterior division Right)

286 voxels covering 13% of atlas.Cereb8 r (Cerebelum 8 Right)

282 voxels covering 100% of atlas.HG r (Heschl's Gyrus Right)

280 voxels covering 100% of atlas.aSTG l (Superior Temporal Gyrus, anterior division Left)

278 voxels covering 100% of atlas.aSTG r (Superior Temporal Gyrus, anterior division Right)

263 voxels covering 38% of atlas.toITG l (Inferior Temporal Gyrus, temporooccipital part Left)

254 voxels covering 14% of atlas.Cereb8 l (Cerebelum 8 Left)

236 voxels covering 98% of atlas.Ver8 (Vermis 8)

230 voxels covering 100% of atlas.Ver3 (Vermis 3)

204 voxels covering 18% of atlas.toMTG r (Middle Temporal Gyrus, temporooccipital part Right)

200 voxels covering 31% of atlas.SMA L(Juxtapositional Lobule Cortex -formerly Supplementary Motor Cortex- Left)

184 voxels covering 94% of atlas.Ver7 (Vermis 7)

182 voxels covering 100% of atlas.Cereb3 r (Cerebelum 3 Right)

181 voxels covering 23% of atlas.toITG r (Inferior Temporal Gyrus, temporooccipital part Right)

171 voxels covering 8% of atlas.Cereb2 r (Cerebelum Crus2 Right)

166 voxels covering 100% of atlas.Ver9 (Vermis 9)

155 voxels covering 48% of atlas.aITG r (Inferior Temporal Gyrus, anterior division Right)

132 voxels covering 100% of atlas.Cereb3 l (Cerebelum 3 Left)

127 voxels covering 7% of atlas.Cereb2 l (Cerebelum Crus2 Left)

116 voxels covering 43% of atlas.Pallidum r

116 voxels covering 38% of atlas.Pallidum l

104 voxels covering 97% of atlas.Accumbens l

104 voxels covering 99% of atlas.Ver10 (Vermis 10)

95 voxels covering 28% of atlas.aITG l (Inferior Temporal Gyrus, anterior division Left)

84 voxels covering 100% of atlas.Accumbens r

78 voxels covering 12% of atlas.Cuneal r (Cuneal Cortex Right)

58 voxels covering 41% of atlas.SCC r (Supracalcarine Cortex Right)

58 voxels covering 11% of atlas.Caudate l

58 voxels covering 39% of atlas.Cereb10 l (Cerebelum 10 Left)

53 voxels covering 10% of atlas.Cuneal l (Cuneal Cortex Left)

49 voxels covering 67% of atlas.SCC l (Supracalcarine Cortex Left)

48 voxels covering 9% of atlas.Caudate r

47 voxels covering 100% of atlas.Ver12 (Vermis 1 2)

43 voxels covering 27% of atlas.Cereb10 r (Cerebelum 10 Right)

40 voxels covering 5% of atlas.toMTG l (Middle Temporal Gyrus, temporooccipital part Left)

39 voxels covering 4% of atlas.pITG r (Inferior Temporal Gyrus, posterior division Right)

30 voxels covering 5% of atlas.Cereb7 l (Cerebelum 7b Left)

27 voxels covering 3% of atlas.pITG l (Inferior Temporal Gyrus, posterior division Left)

16 voxels covering 3% of atlas.Cereb7 r (Cerebelum 7b Right)

11 voxels covering 2% of atlas.IFG oper r (Inferior Frontal Gyrus, pars opercularis Right)

8 voxels covering 2% of atlas.FO l (Frontal Operculum Cortex Left)

5 voxels covering 2% of atlas.FO r (Frontal Operculum Cortex Right)

2 voxels covering 0% of atlas.IFG tri l (Inferior Frontal Gyrus, pars triangularis Left)

1 voxels covering 0% of atlas.IFG tri r (Inferior Frontal Gyrus, pars triangularis Right)

33233 voxels covering 9% of atlas.not-labeled

**Table S7** Cluster of voxels connected with the left hippocampus

Coordinates -52 -32 -26

Size 1243

671 voxels covering 66% of atlas.pITG l (Inferior Temporal Gyrus, posterior division Left)

48 voxels covering 6% of atlas.pTFusC l (Temporal Fusiform Cortex, posterior division Left)

41 voxels covering 6% of atlas.toITG l (Inferior Temporal Gyrus, temporooccipital part Left)

33 voxels covering 10% of atlas.aITG l (Inferior Temporal Gyrus, anterior division Left)

2 voxels covering 0% of atlas.pMTG l (Middle Temporal Gyrus, posterior division Left)

2 voxels covering 0% of atlas.Cereb1 l (Cerebelum Crus1 Left)

446 voxels covering 0% of atlas.not-labeled

**SUPPLEMENTARY FIGURES**


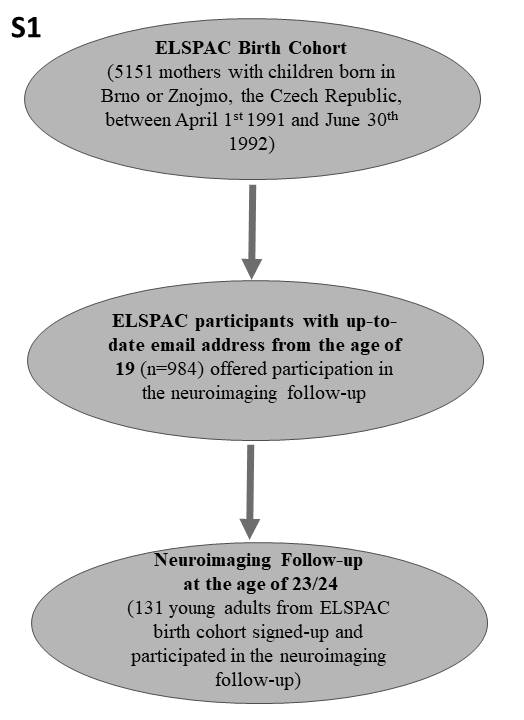


**Figure S1** Recruitment flow diagram


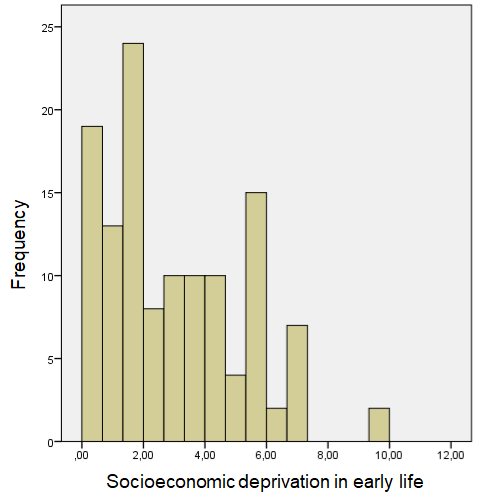


**Figure S2** Distribution of the final early-life socioeconomic deprivation score

**
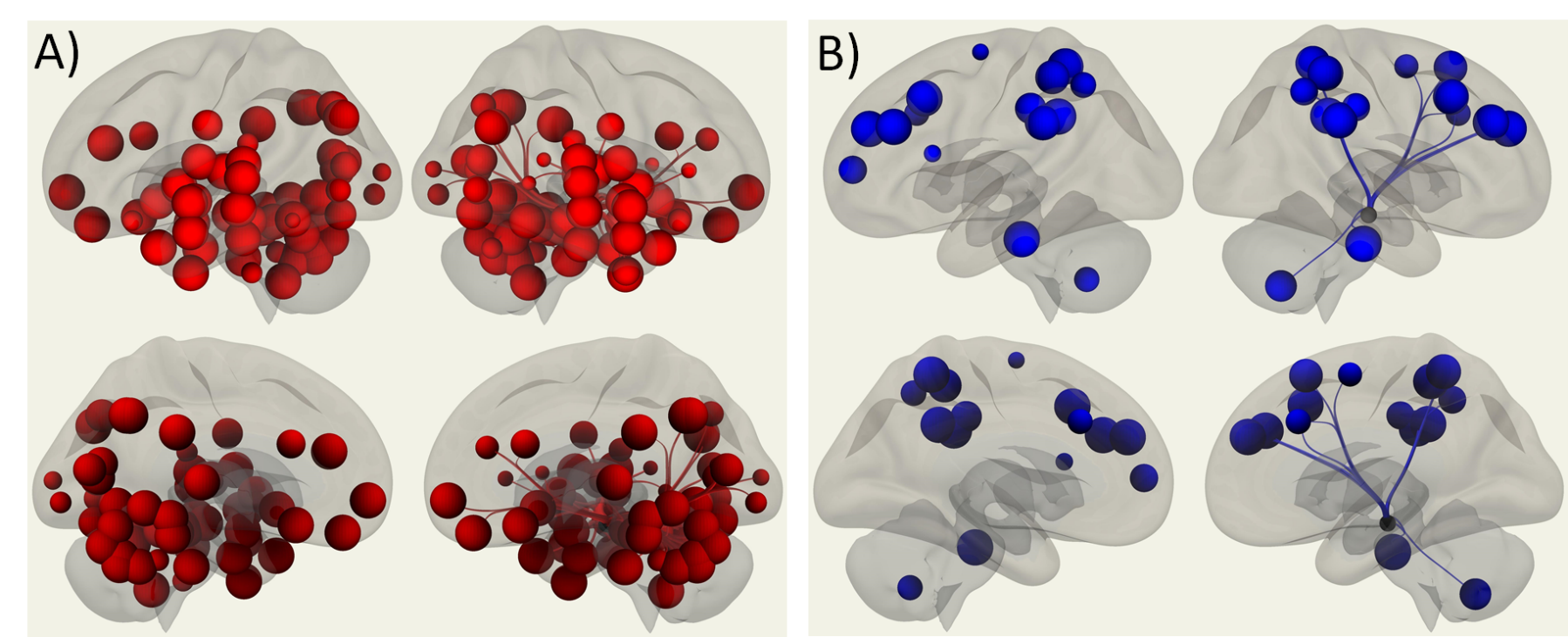
Figure S3** Connectivity between right hippocampus and other regions of interest

A) displays positive connectivity between the right hippocampus and left hippocampus, amygdalae, brain stem, vermis and other parts of cerebellum, many areas in temporal lobe (temporal poles, temporal occipital, middle temporal gyrus, superior temporal gyrus, temporal fusiform, medial occipitotemporal gyrus, transverse temporal gyrus, planum temporale), frontal lobe (frontal medial cortex, medial prefrontal cortex, frontal orbital cortex), parietal lobe (lateral parietal cortex, posterior cingulate cortex, paracingulate gyrus, precuneus), occipital lobe (occipital fusiform cortex, intracalcarice cortex, supracalcarine cortex, lateral occipital, occipital poles), subcallosal cortex, insular cortex and central opercular cortex,

B) displays negative connectivity between the right hippocampus and several areas in frontal lobe (rostral prefrontal cortex, lateral prefrontal cortex, middle frontal gyrus, superior frontal gyrus, left frontal pole, inferior frontal gyrus, frontal eye field), parietal lobe (supramarginal gyrus, superior parietal cortex, intraparietal sulcus, posterior parietal cortex, anterior cingulate cortex), temporal lobe (inferior temporal gyrus) and a small part of cerebellum.

All regions of interest are significantly connected with hippocampus at the level of false discovery rate (FDR) p<0.05.

**References**

**Behzadi, Y., Restom, K., Liau, J. & Liu, T. T.** (2007). A component based noise correction method (CompCor) for BOLD and perfusion based fMRI. *Neuroimage* **37**, 90-101.

**Bobak, M., Pikhart, H., Rose, R., Hertzman, C. & Marmot, M.** (2000). Socioeconomic factors, material inequalities, and perceived control in self-rated health: cross-sectional data from seven post-communist countries. *Social science & medicine* **51**, 1343-1350.

**Cermakova, P., Formanek, T., Kagstrom, A. & Winkler, P.** (2018). Socioeconomic position in childhood and cognitive aging in Europe. *Neurology* **91**, e1602-e1610.

**Cermakova, P., Pikhart, H., Kubinova, R. & Bobak, M.** (2020a). Education as inefficient resource against depressive symptoms in the Czech Republic: cross-sectional analysis of the HAPIEE study. *European Journal of Public Health*.

**Cermakova, P., Pikhart, H., Ruiz, M., Kubinova, R. & Bobak, M.** (2020b). Socioeconomic position in childhood and depressive symptoms in later adulthood in the Czech Republic. *Journal of Affective Disorders*.

**Erikson, R., Goldthorpe, J. H. & Portocarero, L.** (1979). Intergenerational class mobility in three Western European societies: England, France and Sweden. *The British Journal of Sociology* **30**, 415-441.

**Galobardes, B., Shaw, M., Lawlor, D. A., Lynch, J. W. & Davey Smith, G.** (2006a). Indicators of socioeconomic position (part 1). *J Epidemiol Community Health* **60**, 7-12.

**Galobardes, B., Shaw, M., Lawlor, D. A., Lynch, J. W. & Davey Smith, G.** (2006b). Indicators of socioeconomic position (part 2). *J Epidemiol Community Health* **60**, 95-101.

**Ganzeboom, H. B. & Treiman, D. J.** (1996). Internationally comparable measures of occupational status for the 1988 International Standard Classification of Occupations. *Social science research* **25**, 201-239.

**Iglesias, J. E., Augustinack, J. C., Nguyen, K., Player, C. M., Player, A., Wright, M., Roy, N., Frosch, M. P., McKee, A. C. & Wald, L. L.** (2015). A computational atlas of the hippocampal formation using ex vivo, ultra-high resolution MRI: application to adaptive segmentation of in vivo MRI. *Neuroimage* **115**, 117-137.

**Kagstrom, A., Alexova, A., Tuskova, E., Csajbók, Z., Schomerus, G., Formanek, T., Mladá, K., Winkler, P. & Cermakova, P.** (2019). The treatment gap for mental disorders and associated factors in the Czech Republic. *European Psychiatry* **59**, 37-43.

**Mareckova, K., Klasnja, A., Andryskova, L., Brazdil, M. & Paus, T.** (2019a). Developmental origins of depression-related white matter properties: Findings from a prenatal birth cohort. *Hum Brain Mapp* **40**, 1155-1163.

**Mareckova, K., Klasnja, A., Bencurova, P., Andryskova, L., Brazdil, M. & Paus, T.** (2019b). Prenatal Stress, Mood, and Gray Matter Volume in Young Adulthood. *Cereb Cortex* **29**, 1244-1250.

**Mareckova, K., Marecek, R., Andryskova, L., Brazdil, M. & Nikolova, Y. S.** (2020). Maternal Depressive Symptoms During Pregnancy and Brain Age in Young Adult Offspring: Findings from a Prenatal Birth Cohort. *Cerebral Cortex*.

**Mareckova, K., Marecek, R., Bencurova, P., Klanova, J., Dusek, L. & Brazdil, M.** (2018). Perinatal stress and human hippocampal volume: Findings from typically developing young adults. *Sci Rep* **8**, 4696.

**Whitfield-Gabrieli, S. & Nieto-Castanon, A.** (2012). Conn: a functional connectivity toolbox for correlated and anticorrelated brain networks. *Brain connectivity* **2**, 125-141.

**Winkler, P., Formánek, T., Mladá, K. & Cermakova, P.** (2018). The CZEch Mental health Study (CZEMS): Study rationale, design, and methods. *International journal of methods in psychiatric research* **27**, e1728.
